# Supplementary material for: Coordinate regulation of mutant NPC1 degradation by selective ER autophagy and MARCH6-dependent ERAD
Source: Nat Commun. 2018 Sep 10;9:3671. doi: 10.1038/s41467-018-06115-2 (PMC6131187; doi:10.1038/s41467-018-06115-2)
Supplement: Supplementary file 1 — Supplementary Information [file 41467_2018_6115_MOESM1_ESM.pdf]

Supplementary Information:

Coordinate regulation of mutant NPC1 degradation by selective ER autophagy and MARCH6-dependent ERAD

Schultz et. al.

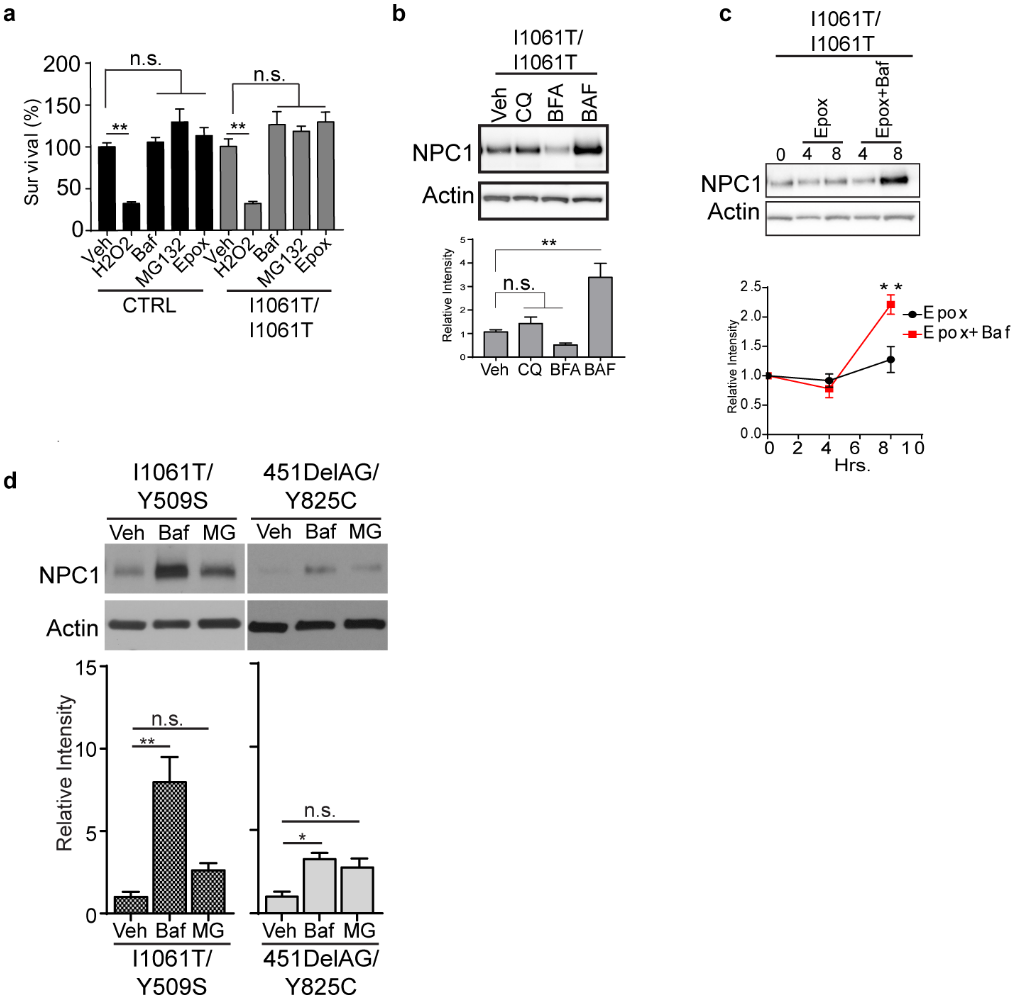

Supplementary Figure 1. Effects of inhibitors on NPC1 protein levels and cell viability.

(a) Primary human fibroblasts expressing WT or I1061T NPC1 were treated with vehicle (Veh), 100nM bafilomycin A1 (Baf), 10 µM MG132 (MG), 100 nM epoxomicin (Epox), or 1000 µM hydrogen peroxide (H_2O_2) for 24 hours, and viability was tested using XTT assay.

(b) I1061T fibroblasts were treated for 24 hours with vehicle (Veh), 10 µM chloroquine (CQ) or 100 nM bafilomycin A1 (Baf), or for 6 hours with 10 µg/ml Brefeldin A (BFA). NPC1 levels were analyzed by western blot. Quantified below.

(c) I1061T fibroblasts were incubated for 0, 4, or 8 hours with 100 nM Epox or with 100 nM Epox + 100 nM Baf. NPC1 protein was analyzed by western blot. Quantified below.

(d) Patient fibroblasts with the indicated *NPC1* mutations were treated for 24 hours with vehicle, 100 nM Baf, or 10 μM MG. Quantified below.

(a, b, c, d) Data are mean ± s.e.m. from three independent experiments. n.s., not significant, $*p \leq 0.05$, $**p \leq 0.01$, $***p \leq 0.001$, $****p \leq 0.0001$. (a, b, c, d) ANOVA with (a, b, c) Tukey and (c) Bonferonni posthoc. a: ($n=3$, $df=9$, $F=14.89$), b: ($n=3$, $df=3$, $F=26.07$), c: ($n=3$, $df=2$, $F=15.24$), d: ($n=3$, $df=2$, $F=7.76$).

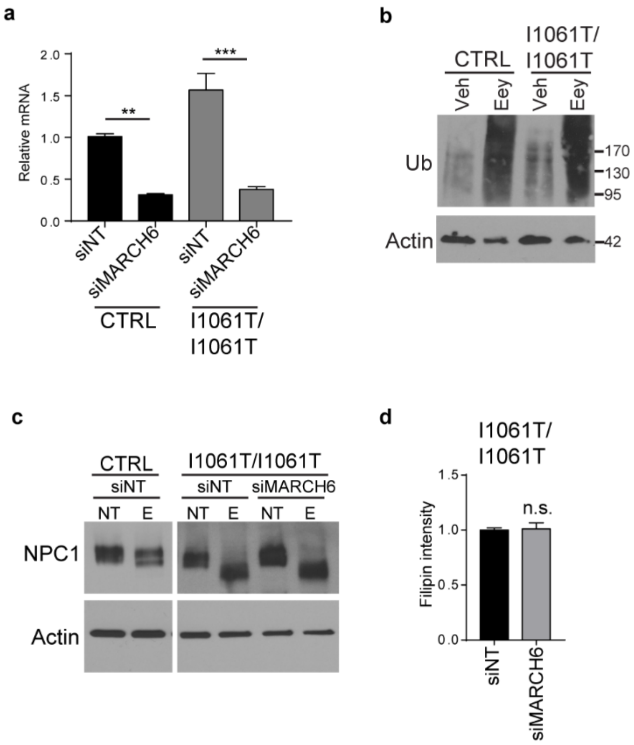

Supplementary Figure 2. MARCH6 knockdown does not alter I1061T trafficking, and Eeyarestatin I accumulates high molecular weight ubiquitinated species.

(a, c, d) Primary fibroblasts expressing WT or I1061T NPC1 were treated with non-targeting (NT) or MARCH6 siRNA at t=0 and t=24 hours. MARCH6 mRNA expression (normalized to GAPDH) was analyzed by qPCR. One-Way ANOVA with Tukey post hoc ($F=33.01$, $df=3$, $n=3$), $^{**}p \leq 0.01$, $^{***}p \leq 0.001$.

(b) Primary fibroblasts expressing WT or I1061T NPC1 were treated with vehicle (Veh) or 10 μM eeyarestatin I (Eey) for 16 hours, and lysates were analyzed for ubiquitin by western blot.

(c) Lysates were digested with endoglycosidase H (E) or not treated (NT) and then analyzed by western blot.

(d) I1061T cells were stained by filipin. Student's t-test ($df= 4$, $n=3$, $t=.1868$), n.s.= not significant.

Data are mean ± s.e.m. from 3 experiments.

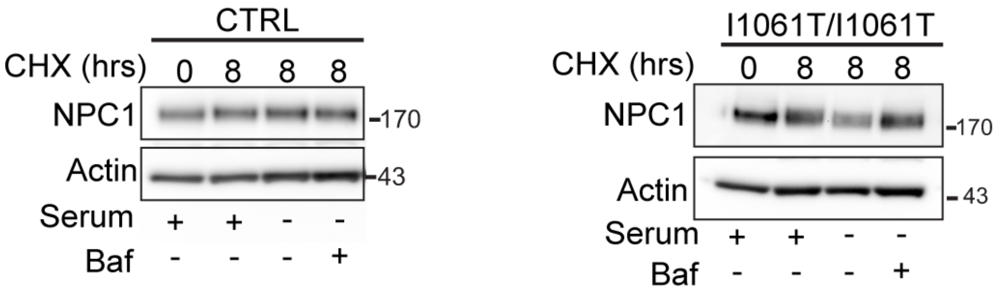

Supplementary Figure 3. Serum Starvation increases I1061T degradation.

Primary fibroblasts expressing WT or I1061T NPC1 were treated with 60 μg/ml cycloheximide (CHX) for the indicated times in the presence or absence of serum. Treatment with 100 nM Bafilomycin (Baf) during serum starvation recovered I1061T protein levels.

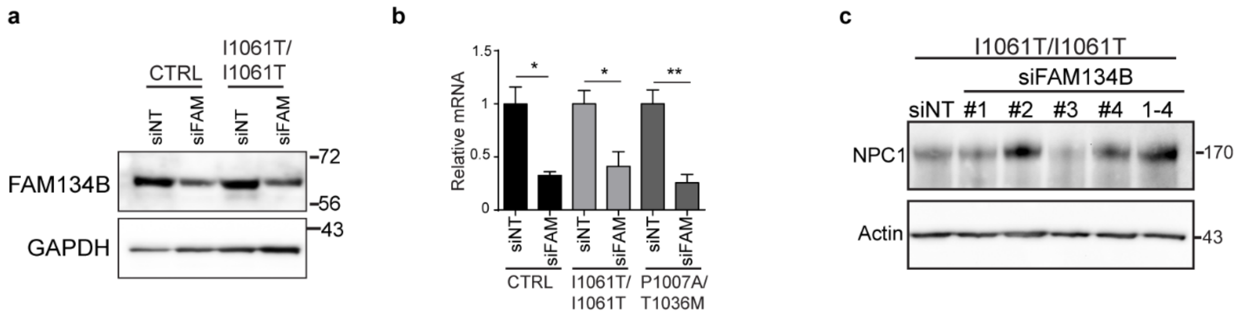

Supplementary Figure 4. FAM134B mRNA knockdown by targeted siRNA.

(a, c) CTRL, I1061T, or (b) P1007A/T1036M *NPC1* fibroblasts were treated with non-targeting (NT) or FAM134B (FAM) smartpool siRNAs at t=0 and t=24 hours. FAM134B knockdown was analyzed by (a) western blot or (b) mRNA expression (normalized to GAPDH) by qPCR at t=48 hours. (b) Data are mean ± s.e.m. from 3 experiments. One-way ANOVA with Tukey post hoc (F=9.6, df=5, n=3), *p≤0.05, **p≤0.01.

(c) To determine which FAM134B siRNAs in the SMARTpool contribute to I1061T accumulation, I1061T fibroblasts were treated as above with individual siRNA from the pool, as follows: #1, #2, #3, #4, or SMARTpool (1-4) containing all four siRNAs. This revealed FAM134B siRNAs #2 and #4 accumulate I1061T protein. NT, non-targeting siRNA.

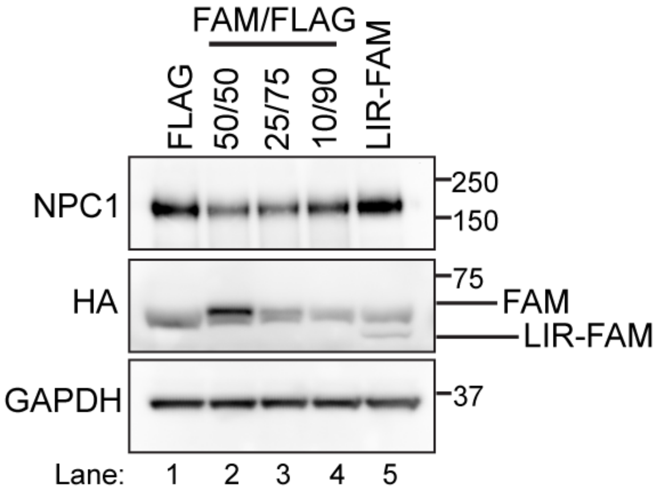

Supplementary Figure 5: FAM134B lacking LIR domain does not alter I1061T NPC1 levels. To confirm LIR-FAM-HA over-expression does not alter I1061T NPC1 despite expression differences vs. WT-FAM, I1061T/I1061T fibroblasts were transfected with plasmids as follows: 1) 1.5 µg FLAG (control vector), 2) 0.75 µg FLAG + 0.75 µg FAM134B-HA, 3) 1.125 µg FLAG + 0.375 µg FAM134B-HA, 4) 1.35 µg FLAG + 0.15 µg FAM134B-HA, 5) 1.5 µg LIR-FAM134B-HA. Cell lysates were collected 48 hours post-transfection and analyzed by western blot.

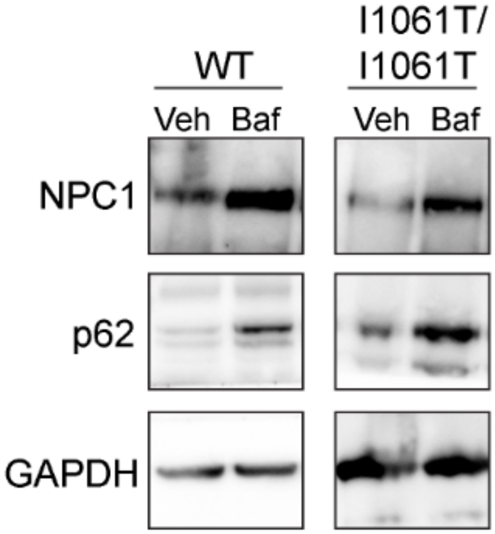

Supplementary Figure 6. NPC1 protein accumulates in brain slices after treatment with bafilomycin A1.

Cultured brain slices from 4 week old I1061T/I1061T and WT mice were treated with vehicle (Veh) or 5 mM Baf for 48 hours. Equal amounts of protein lysates were resolved by SDS-PAGE. Membranes were blotted for NPC1, p62, and GAPDH. Baf treatment increased p62 and NPC1 protein levels.

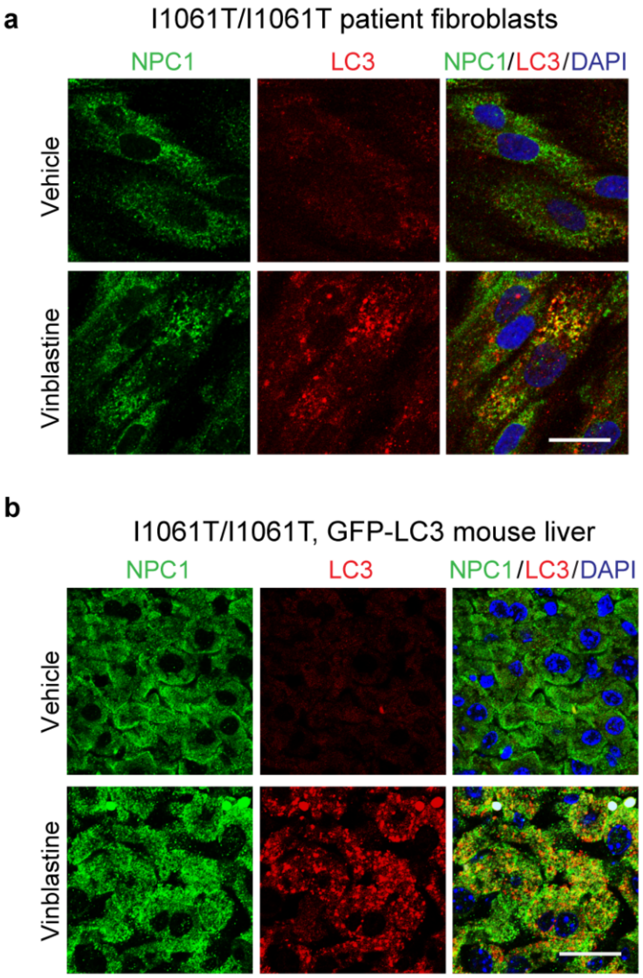

Supplementary Figure 7. Vinblastine induces I1061T NPC1 and LC3 co-localization.

(a) I1061T NPC1 human fibroblasts were incubated with vehicle or 50 μM vinblastine for two hours.

(b) Seven-week-old I1061T/I1061T-*Npc1*, GFP-LC3 mice were treated with vehicle or vinblastine (.05 mg/g i.p.) for two hours. Liver was harvested for imaging.

NPC1 (green), LC3 (red), and DAPI (blue) were visualized by confocal microscopy. Scale bar=25μM.

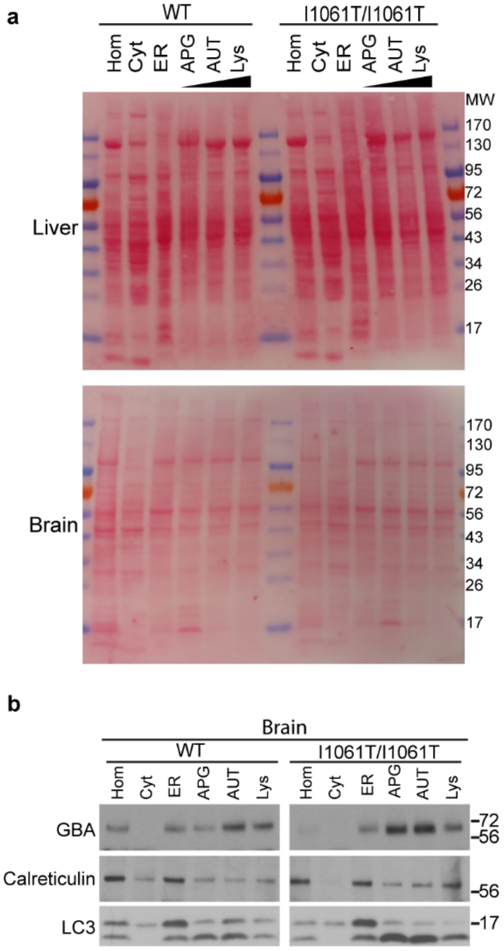

Supplementary Figure 8. Analysis of mouse liver and brain fractions.

Seven-week-old WT or I1061T/I1061T-*Npc1* mice were treated with vinblastine (.05 mg/g i.p.) for two hours. Fractions from Fig. 7b (liver) and Fig. 8a (brain) were resolved on SDS-PAGE. Whole lysates (Hom) or fractions enriched for cytosol (Cyt), ER, autophagosomes (APG), autophagolysosomes (AUT), or lysosomes (Lys) were transferred and stained by (a) Ponceau S or (b) western blot. (a) Quantification of Ponceau S intensity was measured between the 17 and 170 MW bands. (b) Blots were probed for LC3 (autophagic compartments), calreticulin (ER), or glucocerebrosidase (GBA; lysosomes).

Supplementary Figure 9. Uncropped Blots

Fig. 1a

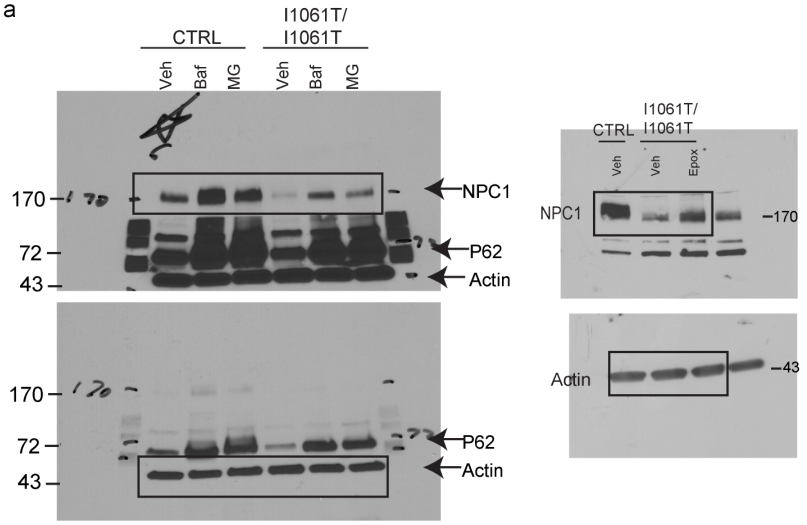

Fig. 1c

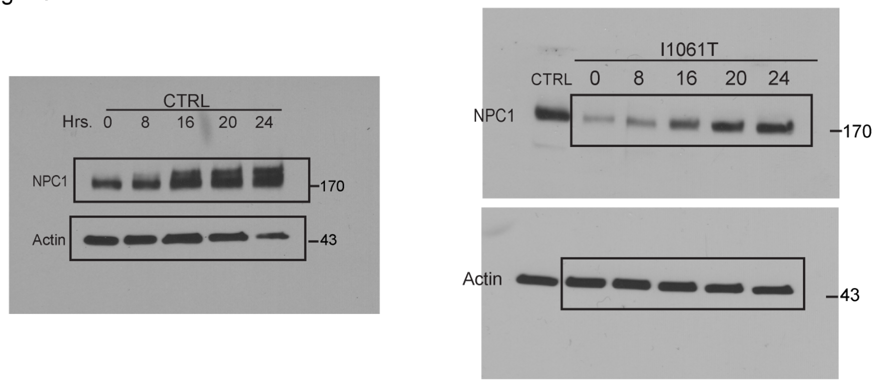

Fig. 1d

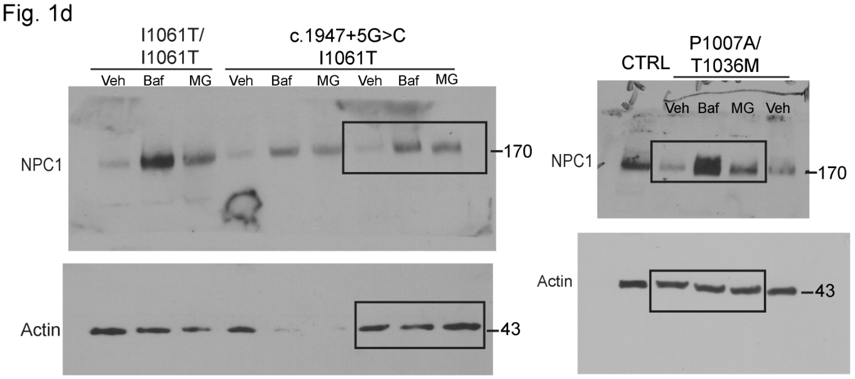

Fig 2a

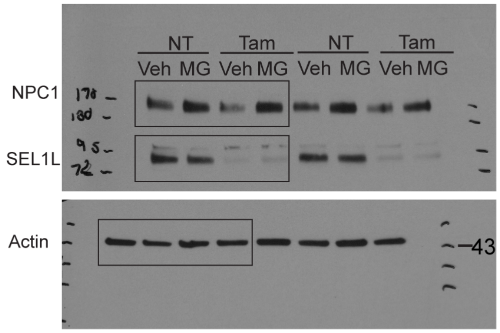

Fig 2b

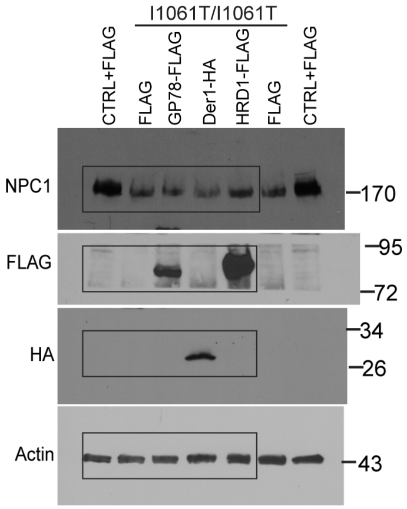

Fig 2c

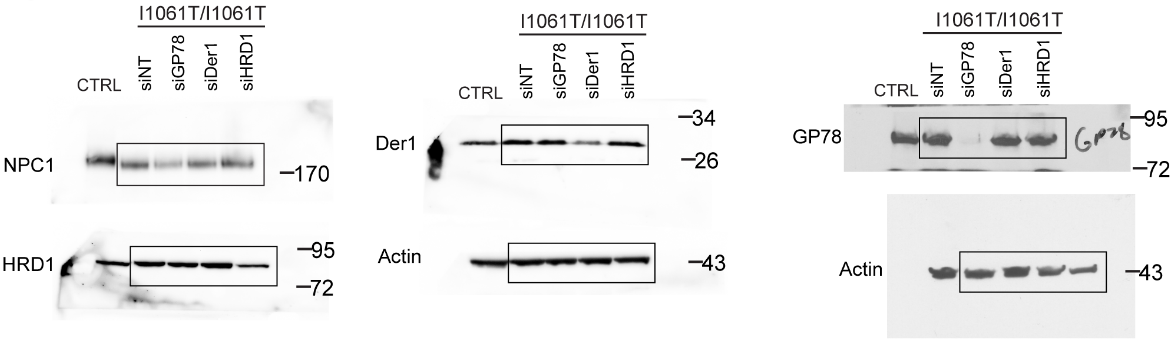

Fig 2d

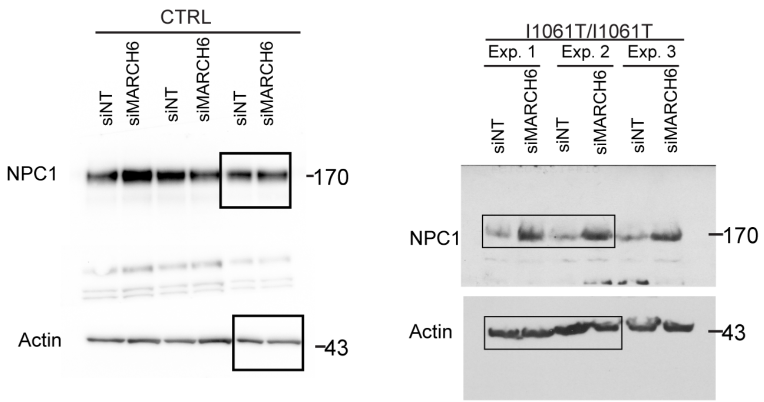

Fig 2e

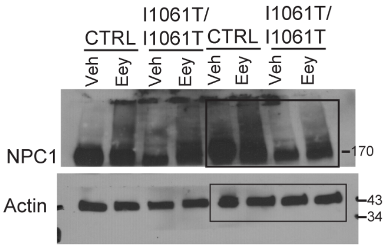

Uncropped Fig. 3b

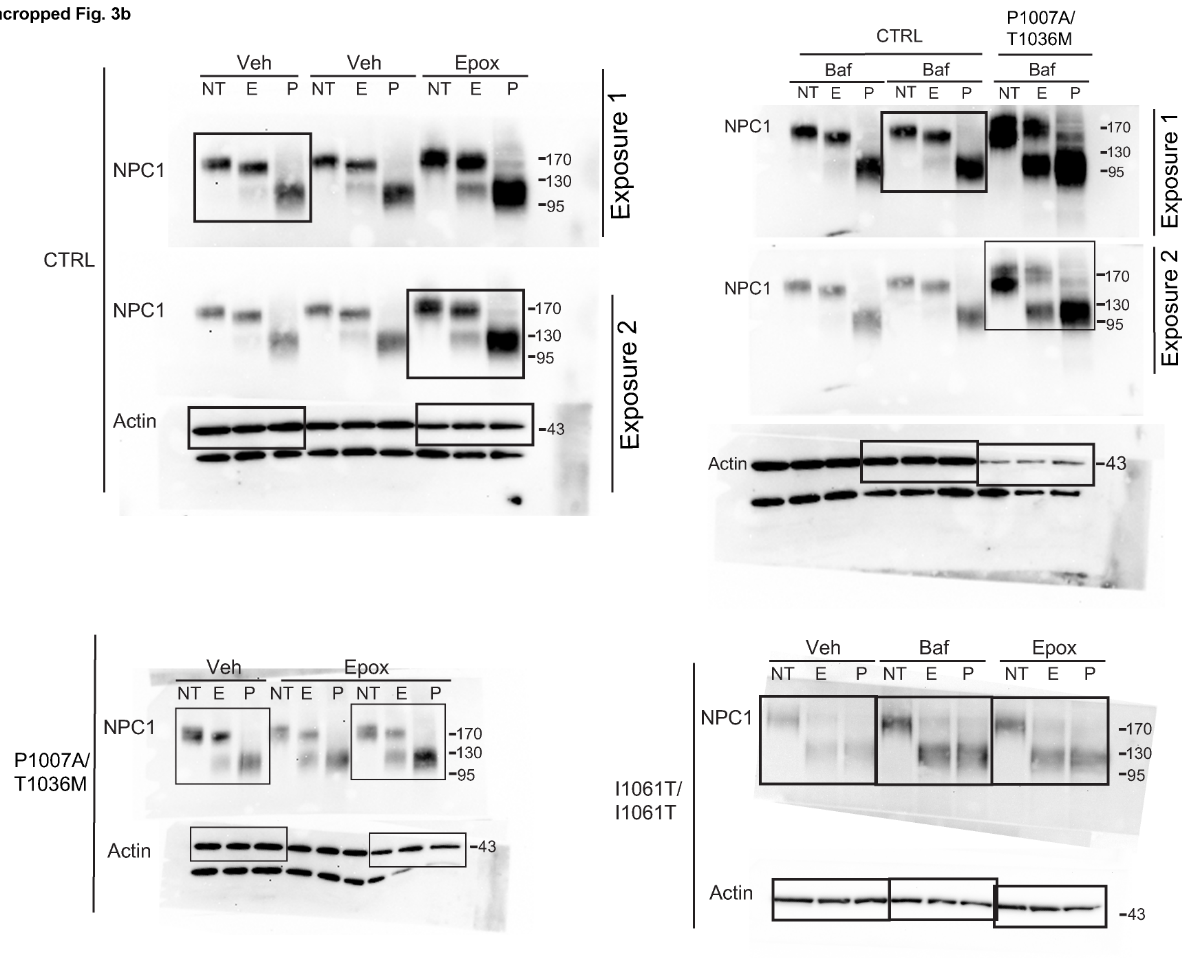

Uncropped Fig. 5a

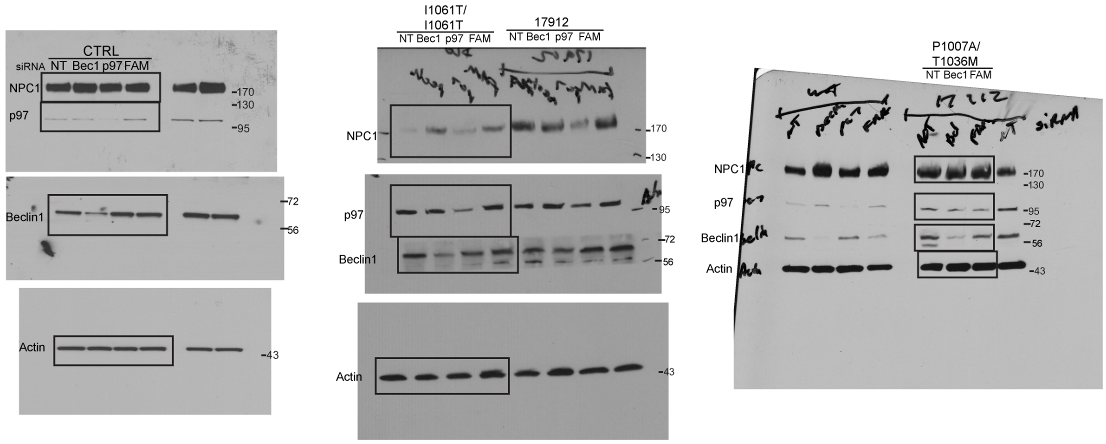

b

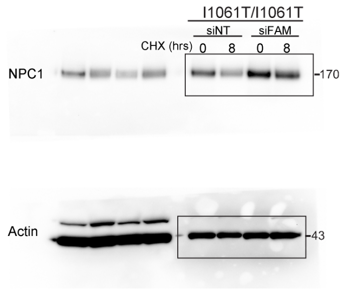

c

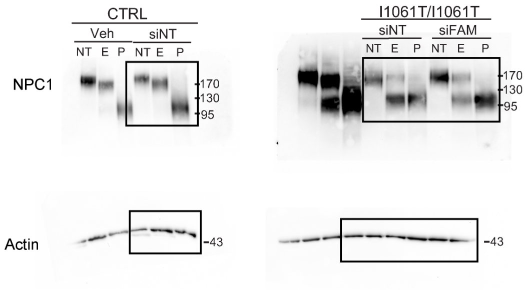

Uncropp Fig. 6a

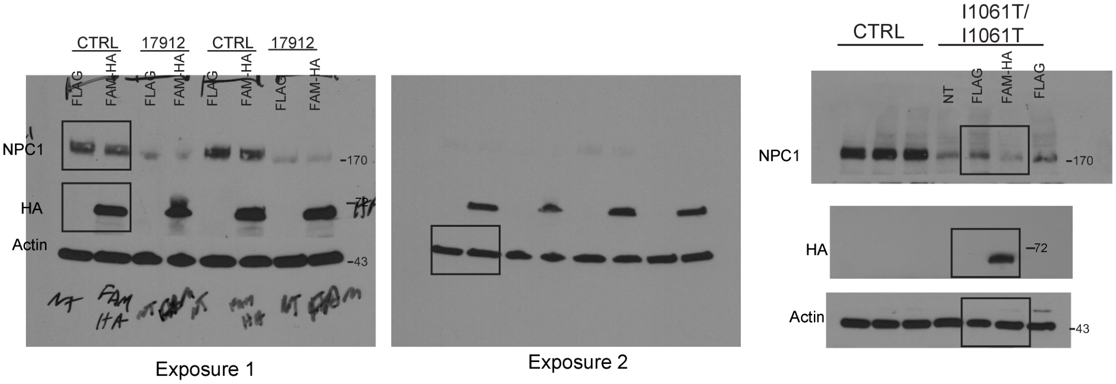

Uncropp Fig. 6c.

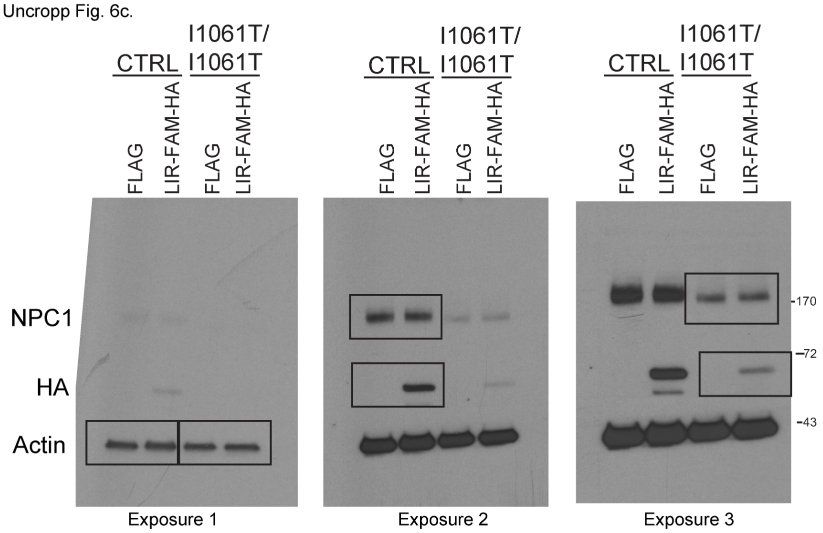

Uncropp Fig. 7b

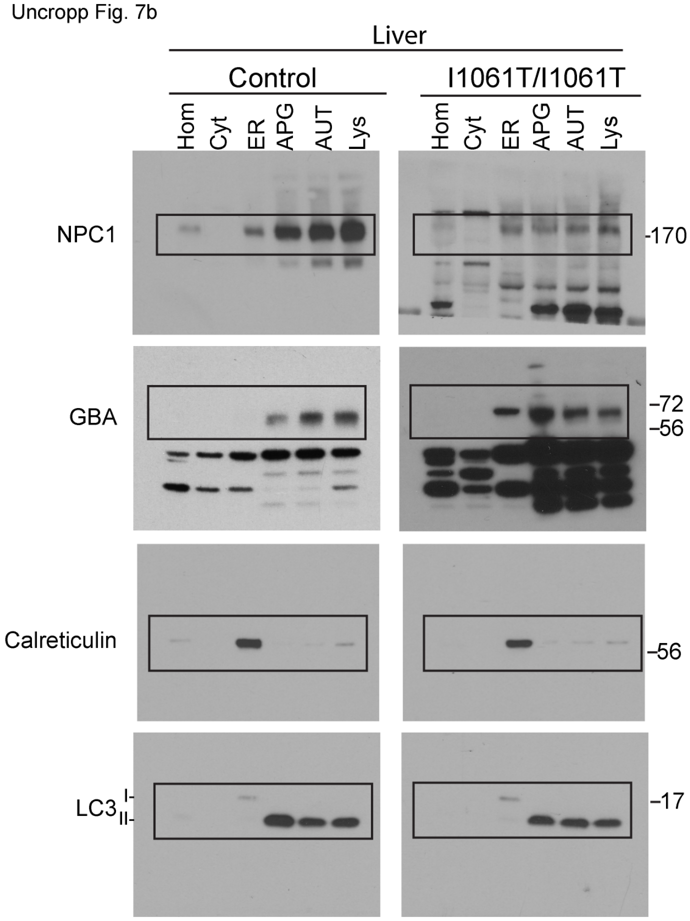

Uncropp Fig. 8a

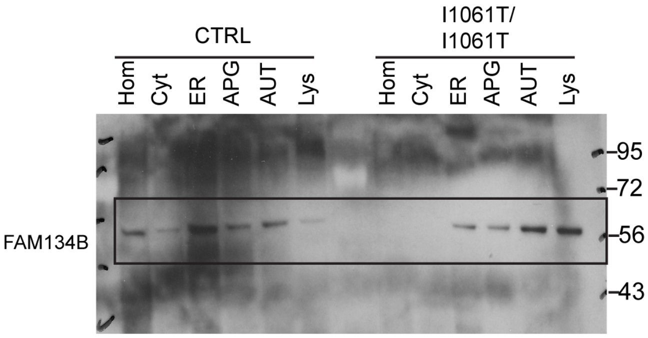

Uncropp Fig. 8c

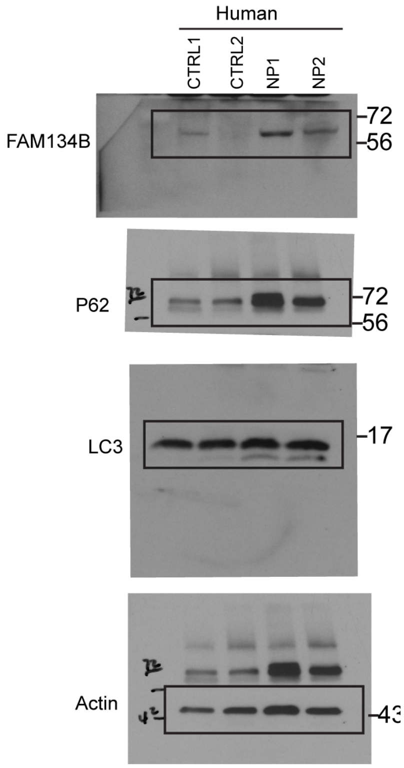

Supplementary Figure 10. Uncropped Supplements

S1

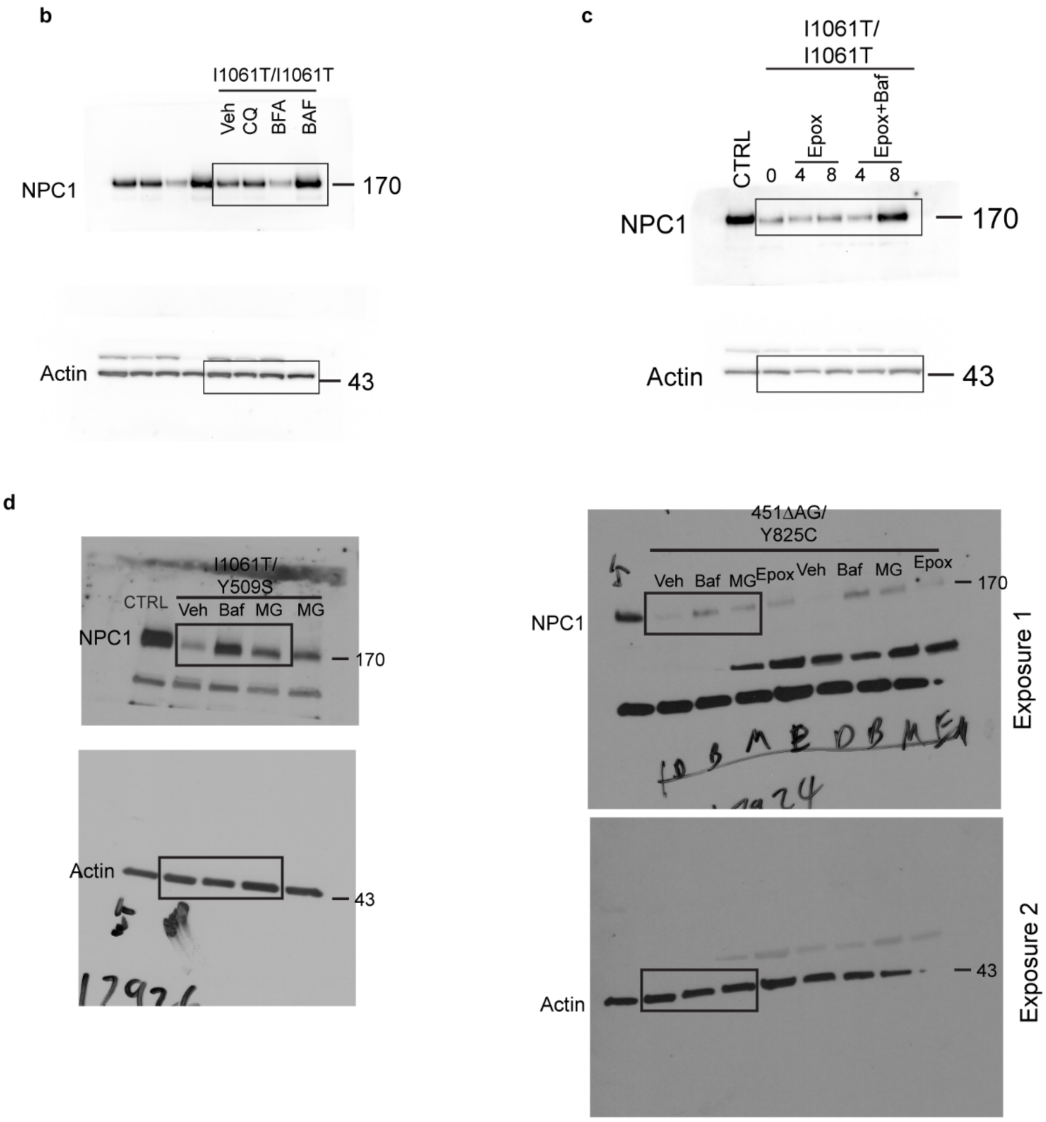

S2

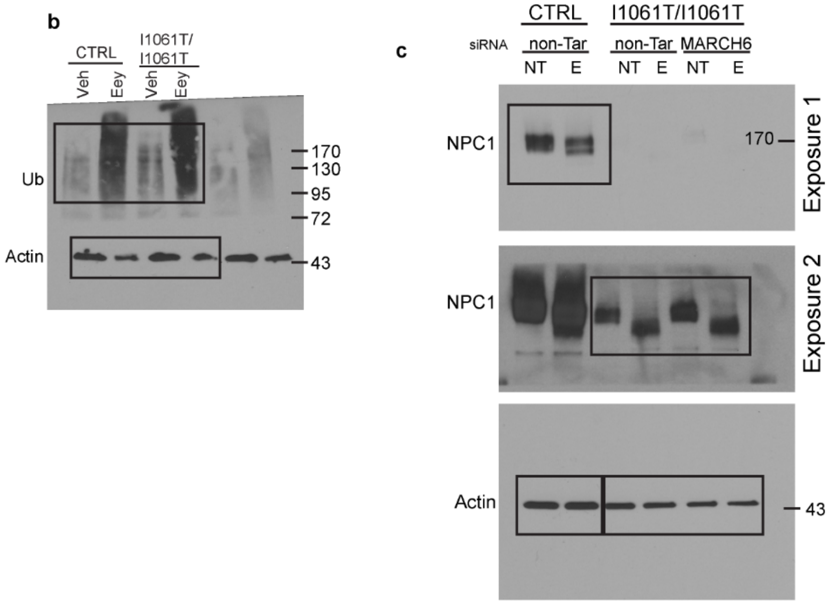
b
CTRL
I1061T/
I1061T
Veh
Eey
Veh
Eey
Ub
Actin
170
130
95
72
43
c
CTRL
I1061T/I1061T
siRNA
non-Tar
non-Tar
MARCH6
NT
E
NT
E
NT
E
NPC1
170
Exposure 1
NPC1
Exposure 2
Actin
43

S3

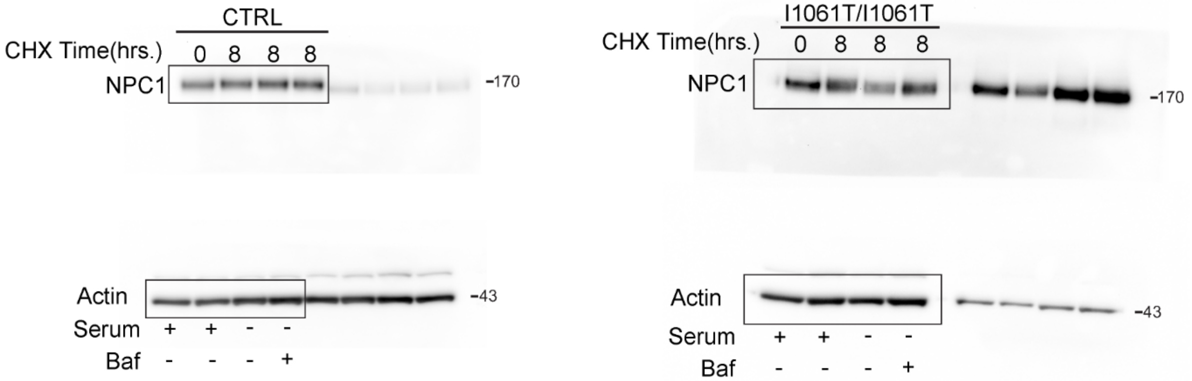
CTRL
CHX Time(hrs.)
0
8
8
8
NPC1
170
Actin
43
Serum
+
+
-
-
Baf
-
-
-
+
I1061T/I1061T
CHX Time(hrs.)
0
8
8
8
NPC1
170
Actin
43
Serum
+
+
-
-
Baf
-
-
-
+

S4

b

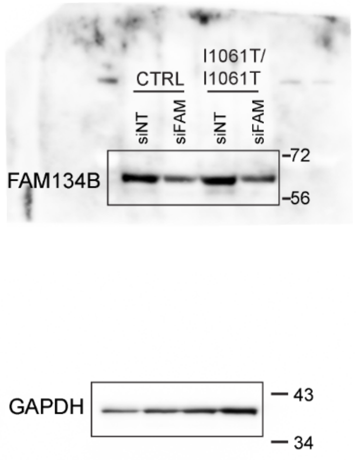
CTRL
I1061T/
I1061T
siNT
siFAM
siNT
siFAM
FAM134B
72
56
GAPDH
43
34

c

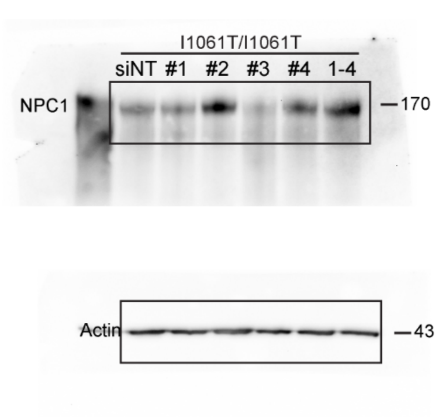
I1061T/I1061T
siNT #1 #2 #3 #4 1-4
NPC1
170
Actin
43

S5

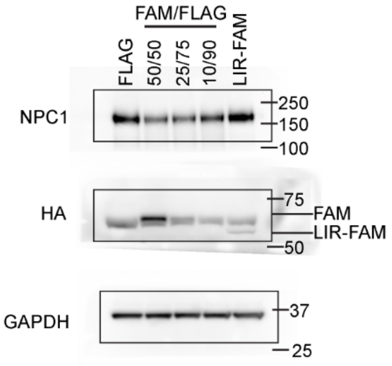
FAM/FLAG
FLAG
50/50
25/75
10/90
LIR-FAM
NPC1
250
150
100
HA
75
FAM
LIR-FAM
50
GAPDH
37
25

S6

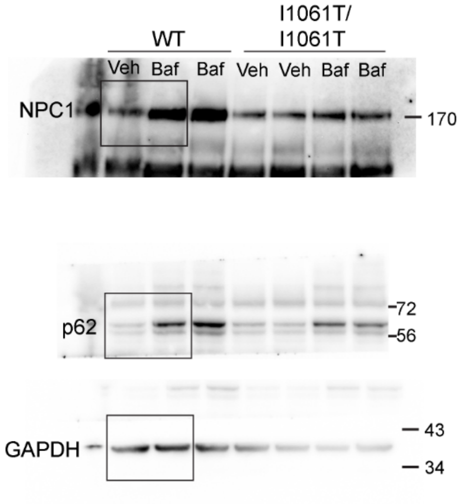
WT
I1061T/
I1061T
Veh Baf Baf Veh Veh Baf Baf
NPC1
170
p62
72
56
GAPDH
43
34

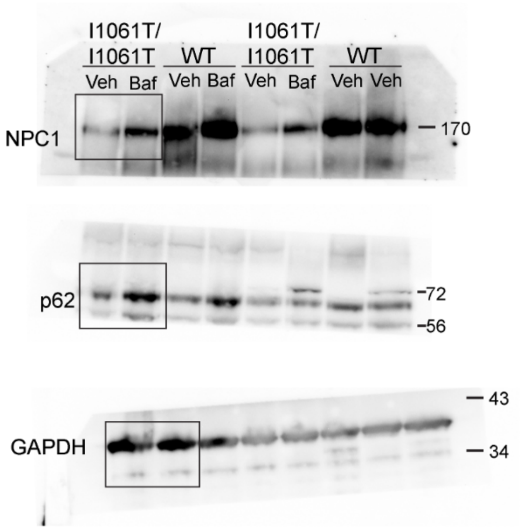
I1061T/
I1061T
WT
I1061T/
I1061T
WT
Veh Baf Veh Baf Veh Baf Veh Veh
NPC1
170
p62
72
56
GAPDH
43
34

S8

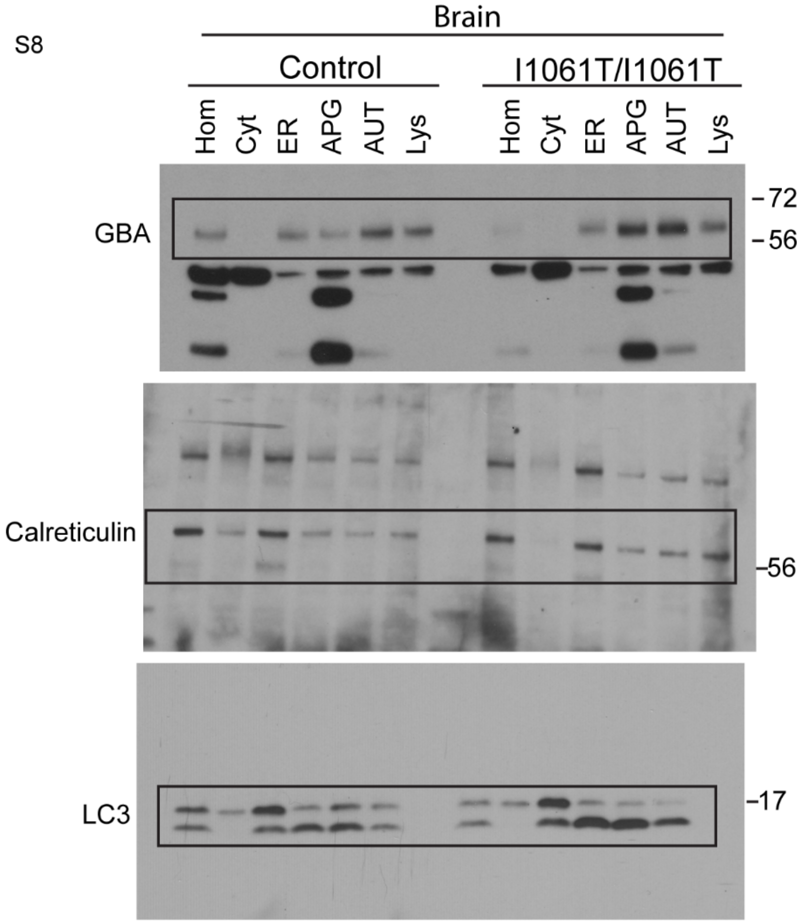
Brain
Control
I1061T/I1061T
Hom
Cyt
ER
APG
AUT
Lys
Hom
Cyt
ER
APG
AUT
Lys
GBA
72
56
Calreticulin
56
LC3
17
